# Retrospective analyses of reasons why children with newly diagnosed type 1 diabetes visit the emergency department

**DOI:** 10.12669/pjms.306.5323

**Published:** 2014

**Authors:** Elif Unsal Avdal, Burcu Arkan, Yasemin Tokem, Medet Korkmaz, Fatma Iltus Kirbiyikoglu

**Affiliations:** 1 Elif Unsal Avdal, RN, PhD, Assistant Professor, Izmir Katip Celebi University, Faculty of Health Sciences, Izmir, Turkey.; 2 Burcu Arkan, RN, PhD, Assistant Professor, Uludag University School of Health, Nursing, Bursa, Turkey.; 3 Yasemin Tokem, RN, PhD, Associate Professor, Izmir Katip Celebi University, Faculty of Health Sciences, Izmir, Turkey.; 4 Medet Korkmaz, RN, PhD, Assistant Professor, Sanko University, Faculty of Health Science,Gaziantep,Turkey.; 5 Fatma Iltus Kirbiyikoglu, MSc, Lecturer, Izmir Katip Celebi University, Faculty of Health Sciences, Izmir, Turkey.

**Keywords:** Type 1 Diabetes Mellitus, Childhood, Emergency Service, Retrospective, Ketoacidosis

## Abstract

***Objectives: ***This retrospective study evaluates the clinical and laboratory values of children with type 1 diabetes at the time of first admission to the emergency service.

***Methods*** *: *It is a retrospective study to examine the clinical & laboratory findings of children visiting the emergency service between 2009 & 2012. The sample of the study included 111 children with newly diagnosed type 1 diabetes. Student t test and chi-square test were used in the analysis.

***Results:*** HbA1c and blood glucose levels and ketoacidosis frequency was found significantly changed (p<0.05). It was detected that the frequency of ketoacidosis at the time of diagnosis was 60% in the children with type 1 diabetes while it was 45% in the second group and 34% in the third group. This difference at the time of admisison was found to be statistically significant (p<0.05).

***Conclusion:*** It became possible to diagnose children with type 1 diabetes much earlier over the course of time, and the number of patients that could be treated before severe diabetic ketoacidosis developed increased. This results is of great importance as it will provide a guide for all medical professionals including nurses.

## INTRODUCTION

In today’s world, diabetes mellitus and other non-contiguous and chronic diseases sharing the same risk factors with it constitute an important health problem. The number of people dying from such chronic diseases as diabetes ranges between 8 & 14 million every year (1-7. The most common type of diabetes seen in childhood is Type 1 diabetes mellitus (Type 1 DM) accompanied by insulin secretion deficiency resulting from pancreatic β-cell damage. Type 1 diabetes results from various degrees of insulin resistance developing in the fatty tissue, liver and skeletal muscle.^1^

It is reported that newly diagnosed type 1 diabetes cases are increasing in many societies and that this increase is seen especially in small children^1^. It is striking that the months when the disease is most commonly seen in children correspond with infection outbreaks. Epidemiological studies in the last 20 years have highlighted dramatic changes in the incidence and prevalence of type 1 diabetes mellitus (DM).^1^^-^^6^ It has been reported that the age of onset has gradually fallen below five while the prevalence rate has increased. Although the reason for this increase is not exactly known, it is mostly attributed to environmental factors.^7^^,^^8^ It has been shown that the number of people visiting hospitals with minor symptoms has increased, that cases of severe ketoacidosis coma has decreased, and that the number of children under the age of five with diabetes has increased with the increase recorded in the number of patients.^9^^,^^10^

In a multi-centre incidence study conducted by the European Working Group on Diabetes in 44 European countries & Israel between 1989 & 1994, the incidence at the age of 15 & below was found to be 3.2/100.000. In China & Venezuela the incidence was found to be 0.1/100.000, and 40/100000 in Finland. Reported incidence in Switzerland, Norway, Portugal, Canada and New Zealand are >20/100000, and incidence is reported to be higher between the ages of 10 and 14. In a multi-centre study conducted in 19 regions in Turkey, the incidence of diabetes in the age range of 0-15 was found to be 2.52/100000.^11^

The clinical course of type 1 diabetes is divided into the phases of acute onset, remission, exacerbation and total diabetes. Polydipsia (drinking an excessive amount of water), polyuria and weight loss are the classic symptoms.^12^ Occasionally, the first symptom may be bedwetting at night in a child who had previously acquired the ability of urine control, or frequent replacement of diapers in an infant, as well as fatigue, nervousness and continuous sleepiness. These symptoms may not be recognized by the family as they appear mild at the onset.^13^^,^^14^ It has been seen in recent cases in which early diagnosis was accomplished that the child/adolescent and the family were not aware of the diabetes or such specific symptoms of diabetes as polydipsia, polyuria and especially weight loss, and visited the hospital because of such accompanying diseases as viral infections, fever, vomiting and diarrhoea, which had suppressed the immune system and increased the insulin requirement; diabetes was incidentally detected when a value exceeding 200 mg/dl was found in the blood glucose measurement. On the other hand, clinical cases that were not detected early frequently during visit to the emergency services due to diabetic ketoacidose (DKA) or coma.^15^^-^^18^

In order to alleviate the burden of diabetes on the individual and family, it should be diagnosed as early as possible and an appropriate treatment should be provided. This study was planned to evaluate the clinical and laboratory values of children with type 1 diabetes at the time of first visit to the emergency service as well as the changes which developed over time with treatment, with the aim of providing a guide for the early diagnosis and treatment of diabetes.

## METHODS

This study was planned as a retrospective study to examine the clinical and laboratory findings of children with newly diagnosed type 1 diabetes applying to the emergency service of the Faculty of Medicine Hospital of Uludag University between 2009 and 2011.

The researcher used a data collection form prepared on the basis of expert opinions as a data collection tool. The form included 14 findings in total to determine age, sex, complaints (polyuria, polydipsia, and weight loss), change in consciousness level, frequency of ketoacidosis, blood glucose, HbA1c, blood gases, duration of intravenous fluid and insulin administration, and sodium potassium level.

***Participants:*** The inclusion criteria for the sample for this retrospective study included; age <19 years, all participants were diagnosed with type 1 diabetes mellitus and discharged with insulin treatment in Hospital of Uludag University Hospital. Hospital records for all subjects were reviewed to study subjects who initially presented with DKA. Demographical data including age, gender, duration of symptoms, clinical features of the disease, presence and severity of DKA at diagnosis were collected. There were no identified cases of type 2 Diabetes Mellitus in this study. The exclusion criteria included any error in hospital records. 

The population of the study consisted of 210 children with newly diagnosed type 1 diabetes. Although the initial plan was to include all these children into the sample, children with type 1 diabetes who had incomplete information in their files and those who were transferred to our hospital after their first treatment was given in another medical centre were excluded from the sample. Thus, the sample of the study included 111 children with newly diagnosed type 1 diabetes. Three groups according to years sampling properties matching determined at the 111 people file through the examination. The data was divided into three groups. In order to evaluate the changes occurring over the course of time, 35 children with type 1 diabetes visiting to the emergency service between 2009 and 2010 were included in the first group, 36 children visiting between 2010 and 2011 were included in the second group, and 40 children visiting between 2011 and 2012 were included in the third group. This analysis was performed according to the sample group.

In addition to such symptoms as polyuria, polydipsia and weight loss, a blood glucose level exceeding 200 mg/dl at the time of diagnosis and HbA1c level were taken as basis for the diagnosis of type 1 diabetes. Children who were diagnosed with obesity, hyperinsulinemia (insulin level >20 IU/ml) and acanthosis nigricans accompanying the general diabetes complaints and who had type 2 diabetes case in their families were diagnosed with type 2 diabetes. A diagnosis of MODY (maturity onset diabetes of youth) was established for patients who approached the hospital with an extra-ketoacidosis milder diabetes who were not obese, and who had low levels of insulin and C-peptide and had early onset diabetes cases below the age of 25 in at least two generations of the family (8-10). Patients diagnosed with type 2 diabetes and MODY were also excluded from the study.

***Procedures: ***Patients receiving treatment for newly diagnosed type 1 diabetes in the institution where the research was conducted were reached through the electronic patient registration system and the protocol book, and data were entered on the data collection Form. Such information as sex, age at the time of diagnosis and complaints was recorded for each patient with the help of an expert opinion specified in the patient files, and information including blood glucose, blood pH and bicarbonate (HCO3) values, HbA1c, sodium and potassium levels, blood osmolarity, duration of intravenous fluid and insulin administration, and time of transition to subcutaneous insulin administration were recorded based on the laboratory findings recorded in the files. Children with type 1 diabetes who had a blood glucose level higher than 300 mg/dl at the time of application, blood pH lower than 7.30 and HCO lower than 315 mEq/L were diagnosed with ketonuria and glycosuria, and those with blood ketone level higher than 3.5 mmol/L were defined as having diabetic ketoacidosis. Patients who suffered from ketonemia and ketouria along with hyperglycaemia but who had blood pH higher than 7.30 and HCO3 over 15 mEq/L were included in the group of patients who visited the hospital without ketoacidosis. All forms of changes in consciousness (lethargy, somnolence, confusion and disorientation) were defined as “consciousness level change”.

***Data collection & analysis: ***Student t test and chi-square test were used in the analysis of the data, and a p value lower than 0.05 was accepted as statistically significant.

***Ethical Considerations: ***Written permission was received from the ethics committee of the Faculty of Medicine of Uludağ University to collect data, and the principle of confidentiality was observed for the patients included in the sample.

## RESULTS

When the children with newly diagnosed type 1 diabetes included in this study were examined in terms of the distribution of age range and sex, no difference was found between the three groups (Table-I).

**Table-I:** Clinical findings in the children with newly diagnosed type 1 diabetes

**Findings**	**1** ^st^ ** Group** **(2009-2010)**	**2** ^nd^ ** Group** **(2010-2011)**	**3** ^rd^ ** Group** **(2011-2012)**	**p value**
Number of children with type 1 diabetes	35	36	40	
Age (year)	9.7±4.2 (2.6-15.8)	9.5±4.3 (1.0-16.0)	9.3±4.5 (1.0-16.0)	p > 0.05
Sex (%)				
Female	17 (48.5)	19 (47.5)	18 (45)	p > 0.05
Male	18 (51.4)	21 (52.5)	22 (55)	
Complaint (%)				p > 0.05
Polyuria	95.7	96.6	95.6	p > 0.05
Polydipsia	92.1	93.5	91.4	p > 0.05
Weight loss	83.4	82.5	81.4	p > 0.05
Consciousness level change (%)	10 (28.5)	4 (10)	2 (5)	p < 0.05

It was detected that polyuria and polydipsia were the most frequent complaints in all of the three groups, followed by weight loss (p>0.05). When patients were examined in terms of consciousness level change, this was found to be 28.5% (n=10) in the first group, 10% (n=4) in the second group and 5% (n=2) in the third group. A statistically significant difference was found between the three groups (p<0.05; Table-I).

**Fig.1:**
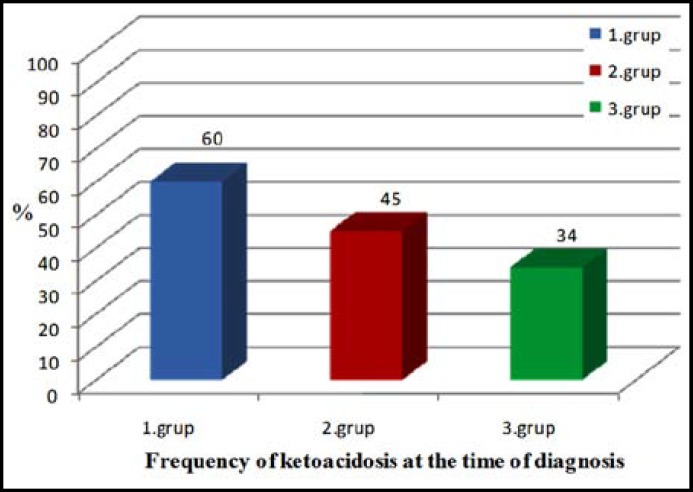
Frequency of ketoacidosis in children with newly diagnosed type 1 diabetes mellitus

We observed that the frequency of ketoacidosis at the time of diagnosis was 60% in the children with type 1 diabetes while it was 45% in the second group and 34% in the third group. This difference at the time of visit was found to be statistically significant (p<0.05) (Fig.1). The rates of those applying to the hospital with the complaints of ketonemia and ketouria accomplying hyperglycaemia without ketoacidosis were determined to be 28.1%, 22.0% and 20.0% in the first, second and third groups respectively.

HbA1c and blood glucose levels at the time of application were found to be significantly lower, while blood pH and bicarbonate levels were found significantly higher, in the second and third groups in comparison to the first group. However, differences in terms of antibody levels were not determined to be statistically significant (Table-II). The average duration from the start of intravenous fluid and insulin administration up to the subcutaneous insulin treatment was found to be higher in the first group than in the second and third groups. The fact that it was lower in the second and third groups is a pleasing finding indicating the success of the treatment (Table-II).

**Table-II:** Laboratory findings of the children with newly diagnosed type 1 diabetes at the time of visit to the emergency service

**Laboratory findings**	**1** ^st^ ** Group** **(2009-2010)**	**2** ^nd^ ** Group** **(2010-2011)**	**3** ^rd^ ** Group** **(2011-2012)**	**p value**
Blood glucose (mg/dl)	410±150.5 (202-850)	385.4±130(156-839)	365.5±110 (140-812)	p< 0.05
HbA1C (%)	14.2±2.4 (9.8-20.8)	12.1±2.3 (7.3-18.2)	11.1±2.3 (7.2-16.2)	p< 0.05
**Blood gases**				
PH	7.16±0.2 (6.80-7.43)	7.30±0.1 (6.89-7.45)	7.40±0.1 (6.89-7.45)	p<0.05
HCO3 (mEq/L)	12.4±7.6 (2.1-25.3)	14.0±6.9 (2.7-28.4)	16.0±6.9 (3.1-28.3)	p<0.05
Duration of intravenous fluid and insulin administration (hour)	13.2±6.6 (0-24)	11.3±6.7 (0-22)	10.3±5.7 (0-21)	p< 0.05
Sodium (mEq/L)	132.9±4.2 (127-143)	135±3.1 (131-144)	136±4.1 (131-144)	p>0.05
Potassium (mEq/L)	4.0±0.5 (3.1-5)	4.1±0.6 (2.8-5.9)	4.3±0.6 (2.8-4.9)	p>0.05

## DISCUSSION

The number of small children with diabetes has recently increased throughout the world along with an increase in the incidence of individuals with type 1 diabetes.^1^ Rosenbauer et al.^10^ detected that the incidence of diabetes among children under the age of five years was 6.86 per 100 000 individuals in 1993 in Germany, rising to 9.68 in 1995. According to the data of the EURODIAB ACE 2001 working group, the annual increase between 1989 & 1994 in the incidence of type 1 diabetes in children in the 0-14 age group in Europe was 3.4%, while the highest increase was reported in children in the 0-4 age group, with a rate of 6.3%. Although the reasons for this increase are not exactly known, it has been attributed not only to genetic factors but also to changes in environmental factors.

The complaints encountered in patients with newly diagnosed type 1 diabetes visiting to the emergency service of hospitals are polyuria, polydipsia and weight loss, and no difference was observed between the three groups in terms of frequency. Similar results have been reported in studies by Al Magamsi and lofs.^19^ However, it was detected that consciousness level change at the time of visit was significantly higher in the first group in comparison to the second and third groups. Whereas the frequency of diabetic ketoacidosis was 60% at the time of visit in the first group, this was found to be 45% in the second group and 34% in the third group. The increase in the number of patients who visit to the hospital only due to hyperglycaemia without acidosis and ketonemia and are diagnosed with diabetes implies that patients with type 1 diabetes are now diagnosed with diabetes in the emergency service much earlier. Diabetic ketoacidosis ranks first among the reasons of death resulting from diabetes in childhood.

The reduced number of patients visiting the hospital with a diagnosis of diabetic ketoacidosis has been interpreted as a positive indication related to decreasing mortality and morbidity. It was reported in a study conducted by Levy-Marchal et al.^4^ at twenty four different centres in Europe that the frequency of diabetic ketoacidosis ranged between 26% and 40% at the first visit, and visit with the complaint of diabetic ketoacidosis is less frequent in countries which have reached high standards of living and a high standard of health services. In parallel to the increase in the rate of early diagnosis among our patients, blood glucose and HbA1c levels were found to be lower and pH and HCO-3 values were found to be higher in the second and third groups at the time of application. Likewise, Jackson et al.^9^ from New Zealand (2001) reported that the frequency of diabetic ketoacidosis was reduced from 63% to 42% in the diabetes patients they monitored over an eight-year period, and blood glucose and HbA1c values at application were found to be significantly lower in a new group in comparison to previous patients. In Iran and in Asia, frequency of DKA was lower than other previous studies which have reported from Asia. Alvi showed that young Asian children in Birmingham had an eightfold increased risk of presenting with DKA as did non–Asian children.^20^

In the phase of emergency treatment, the duration between the start of intravenous fluid and insulin administration and subcutaneous insulin administration decreased dramatically in the patients in the third group. In a study conducted by Bui et al.^16^ (2002) in Australia of the data of diabetic patients in a 15-year period, it was reported that the frequency of DKA was 37.8%, and this frequency did not change over the course of time, with an average duration between intravenous fluid and insulin administrations of 29 hours. It was stated that medical professionals and society should be made more conscious of diabetes in order to secure a decline in the incidence of ketoacidosis in society. This improvement in the emergency treatment requirement of our patients has been interpreted as an expected situation in parallel to the increase in the number of early diagnoses.

***Limitations: ***The limitation of the study is that the data of the patients excluded from the sample due to the lack of information in their files could not be reviewed.

## CONCLUSIONS

According to the results of our study, it became possible to diagnose children with type 1 diabetes much earlier over the course of time, and the number of patients that could be treated before severe diabetic ketoacidosis developed increased. This results in a decrease in morbidity and mortality rates as well as achieving more favourable outcomes in emergency treatment. It is thought that early diagnosis and treatment opportunities will increase as long as the society and the medical professionals become more aware, and the number of accessible medical centres rises and socio-economical opportunities increase.

## Notes

***Clinical practice: ***Findings of this study are of great importance as it will provide a guide for all medical professionals including nurses in the early diagnosis and treatment of type 1 diabetes in the future. Data obtained through this study will ensure that the nursing interventions are planned prospectively for newly diagnosed type 1 diabetes progress in the emergency services more systematically.
